# The 203 kbp Mitochondrial Genome of the Phytopathogenic Fungus *Sclerotinia borealis* Reveals Multiple Invasions of Introns and Genomic Duplications

**DOI:** 10.1371/journal.pone.0107536

**Published:** 2014-09-12

**Authors:** Andrey V. Mardanov, Alexey V. Beletsky, Vitaly V. Kadnikov, Alexander N. Ignatov, Nikolai V. Ravin

**Affiliations:** Centre “Bioengineering”, Russian Academy of Sciences, Moscow, Russia; University of Toronto, Canada

## Abstract

Here we report the complete sequence of the mitochondrial (mt) genome of the necrotrophic phytopathogenic fungus *Sclerotinia borealis*, a member of the order *Helotiales* of Ascomycetes. The 203,051 bp long mtDNA of *S. borealis* represents one of the largest sequenced fungal mt genomes. The large size is mostly determined by the presence of mobile genetic elements, which include 61 introns. Introns contain a total of 125,394 bp, are scattered throughout the genome, and are found in 12 protein-coding genes and in the ribosomal RNA genes. Most introns contain complete or truncated ORFs that are related to homing endonucleases of the LAGLIDADG and GIY-YIG families. Integrations of mobile elements are also evidenced by the presence of two regions similar to fragments of inverton-like plasmids. Although duplications of some short genome regions, resulting in the appearance of truncated extra copies of genes, did occur, we found no evidences of extensive accumulation of repeat sequences accounting for mitochondrial genome size expansion in some other fungi. Comparisons of mtDNA of *S. borealis* with other members of the order *Helotiales* reveal considerable gene order conservation and a dynamic pattern of intron acquisition and loss during evolution. Our data are consistent with the hypothesis that horizontal DNA transfer has played a significant role in the evolution and size expansion of the *S. borealis* mt genome.

## Introduction

The phytopathogenic fungus *Sclerotinia borealis* Bubak & Vleugel, which is an ascomycete belonging to the family *Sclerotiniaceae* of the order *Helotiales*, has a broad host range and causes diseases in at least 17 plant genera from the families *Alliaceae*, *Asteraceae*, *Brassicaceae*, *Campanulaceae*, *Fabaceae*, *Iridaceae*, *Pinaceae*, and *Poaceae* (notably wheat and corn). *S. borealis* is a psychrophilic necrotrophic fungus with an optimum growth temperature between 4°C and 10°C [1]. The main specific feature of the disease called “snow mould” is a white mycelium and sclerotia growth on dead plant tissues [2]. *S. borealis* is distributed mostly in northern regions (Japan, North America, Scandinavia, and Russia). However, the biology of *S. borealis* and its phylogenetic relationships to the other species of *Sclerotiniaceae* are still poorly understood.

Mitochondrial (mt) genomes have been successfully used in evolutionary biology and systematic studies [3], [4], since they evolve faster than nuclear genomes [5], [6]. Fungal mt genomes range in size from about 19 kbp (*Hanseniaspora uvarum*) to 235,849 bp for *Rhizoctonia solani* AG-3 strain Rhs1AP [7], and usually contain 14 genes that encode oxidative phosphorylation system proteins, the large (*rnl*) and small (*rns*) ribosomal RNA subunits, and a fairly constant set of tRNAs genes [8], [9]. Besides this core set of genes, a varying number of introns, often including GIY-YIG or LAGLIDADG endonuclease genes, have been reported [10], [11], [12], [13].

In recent years, the number of complete filamentous fungal mt genome sequences has significantly increased [14], [15], facilitating evolutionary and systematic studies [16], [17], [18], [19], [20], [21], [22]. Currently more than 100 complete fungal mitochondrial genomes are available, but only nine represent the order *Helotiales* of ascomycetes (*Phialocephala subalpina*, *Sclerotinia sclerotiorum*, *Botrytis cinerea* (teleomorph *Botryotinia fuckeliana*), *Glarea lozoyensis*, *Marssonina brunnea,* and four *Rhynchosporium* species) [16], [21], [22]. Only two mitochondrial genomes belong to members of the *Sclerotiniaceae* family, *B. fuckeliana* and *S. sclerotiorum*.

Comparative analysis of mtDNAs of three helotialean species, *P. subalpina*, *B. fuckeliana*, and *S. sclerotiorum*, revealed conservation of core mt genes, but high variability of mtDNA size, which ranged from 43,742 bp (*P. subalpina*) to 128,852 bp (*S. sclerotiorum*) [16]. Considerable variations of mtDNA size were observed even among species of a single genus, *Rhynchosporium*, from 49,539 bp (*Rhynchosporium orthosporum*) to 69,581 bp (*Rhynchosporium commune*) [22]. The large size variations of mt genomes between different fungal species mainly result from the presence or absence of large intronic and intergenic sequences [23], [24], [25], [26]. How the origin and direction of intron acquisition and loss are determined remains poorly understood. One hypothesis suggests that introns were abundant in the ancestral mt genes, but have subsequently been lost in most lineages [27]. Although this hypothesis is currently being discussed [28], both intron loss and gain events are required to explain the uneven distribution of introns across even rather closely related lineages.

In this paper, we report the complete nucleotide sequence of the mt genome of *S. borealis* strain F-4128. We describe the gene content, the genome organisation of the mitochondrial genome of *S. borealis*, and a comparative analysis of the known mt genomes of helotialean fungi. The main focus of this work is on genomic duplications and mobile genetic elements, such as introns and plasmid-related sequences. The distributions and potential origins of these elements are discussed.

## Materials and Methods

### Sequencing, assembly, and annotation of the mitochondrial genome

*S. borealis* F-4128 was obtained from the All-Russia Collection of Microorganisms (VKM). For DNA extraction, mycelia and sclerotia collected from the surface of agar were used. Total DNA was isolated by the SDS-CTAB method [29].

Mt genome sequencing was performed using a total genomic DNA sample without prior isolation of the mtDNA. The genome was sequenced with a Roche Genome Sequencer (GS) FLX, using the XL+ protocol for a shotgun genome library. The GS FLX run resulted in the generation of about 811 MB of sequences with an average read length of 510 bp. The GS FLX reads were assembled into contigs using Newbler Assembler 2.8 (454 Life Sciences, Branford, CT). Two contigs, 190,990 bp (coverage 59X) and 10,928 bp (coverage 62X), were identified as representing the mtDNA on the basis of extensive sequence similarity to known fungal mt genomes. The gaps between the contigs were closed by sequencing of corresponding PCR fragments.

The MFannot tool (http://megasun.bch.umontreal.ca/cgi-bin/mfannot/mfannotInterface.pl) with default settings was used for mt genome annotation, which was adjusted manually by sequence alignment of deduced genes with their intron-less orthologs from related species. Putative proteins encoded by dubious ORFs were analysed by a BLAST homology search against the NCBI protein database. The codon frequency was determined using CodonW (http://www.molbiol.ox.ac.uk/cu/culong.html#Codonw) for catenated ORFs for all protein-coding genes in the *S. borealis* mt genome.

Repeated sequences were identified by a BLASTN search of mt DNA against itself; matches with E- values <e^-3^ were taken into account.

The complete sequence of the mitochondrial genome of *S. borealis* F-4128 has been deposited in GenBank under the accession no. KJ434027.

### Genome comparison and phylogenetic analysis

Whole mtDNA comparisons for the *Peltigerales* and *Helotiales* species *Peltigera malacea*, *Peltigera membranacea*, *P. subalpina*, *B. fuckeliana*, *S. sclerotiorum*, and *S. borealis* were performed using MAUVE 2.3.1 software [30]. The locally collinear blocks identified by MAUVE were compared with the annotated gene features.

For the phylogenetic analysis, we used 14 mitochondrial proteins, including subunits of the respiratory chain complexes (*cox1, cox2, cox3*, and *cob*), ATPase subunits (*atp6, atp8*, and *atp9*), and seven NADH dehydrogenase subunits (*nad1*, *nad2*, *nad3*, *nad4*, *nad4L*, *nad5*, and *nad6*). The list of 51 fungal species used to construct phylogenetic trees is shown in Table S3. Multiple sequence alignment was performed using the MUSCLE program of the MEGA5 [31] package, and poorly aligned positions and gap positions were removed with trimAl [32]. We used RAxML v7.6.6 to calculate the maximum likelihood phylogenetic tree with a gamma model of rate heterogeneity (four discrete rate categories and an estimated alpha parameter) and the WAG substitution matrix. We conducted 500 bootstrap replicates to define the support values on the tree. Also, we constructed a tree with a Bayesian method that produced a topology similar to the ML tree; bayesian analysis was performed using PhyloBayes with a JTT substitution model (4 discrete categories); trees were sampled using every 2 out of 638 generations; the first 150 trees were discarded as burn-in.

## Results and Discussion

### General features of the mt genome of *S. borealis*

**Figure 1:**
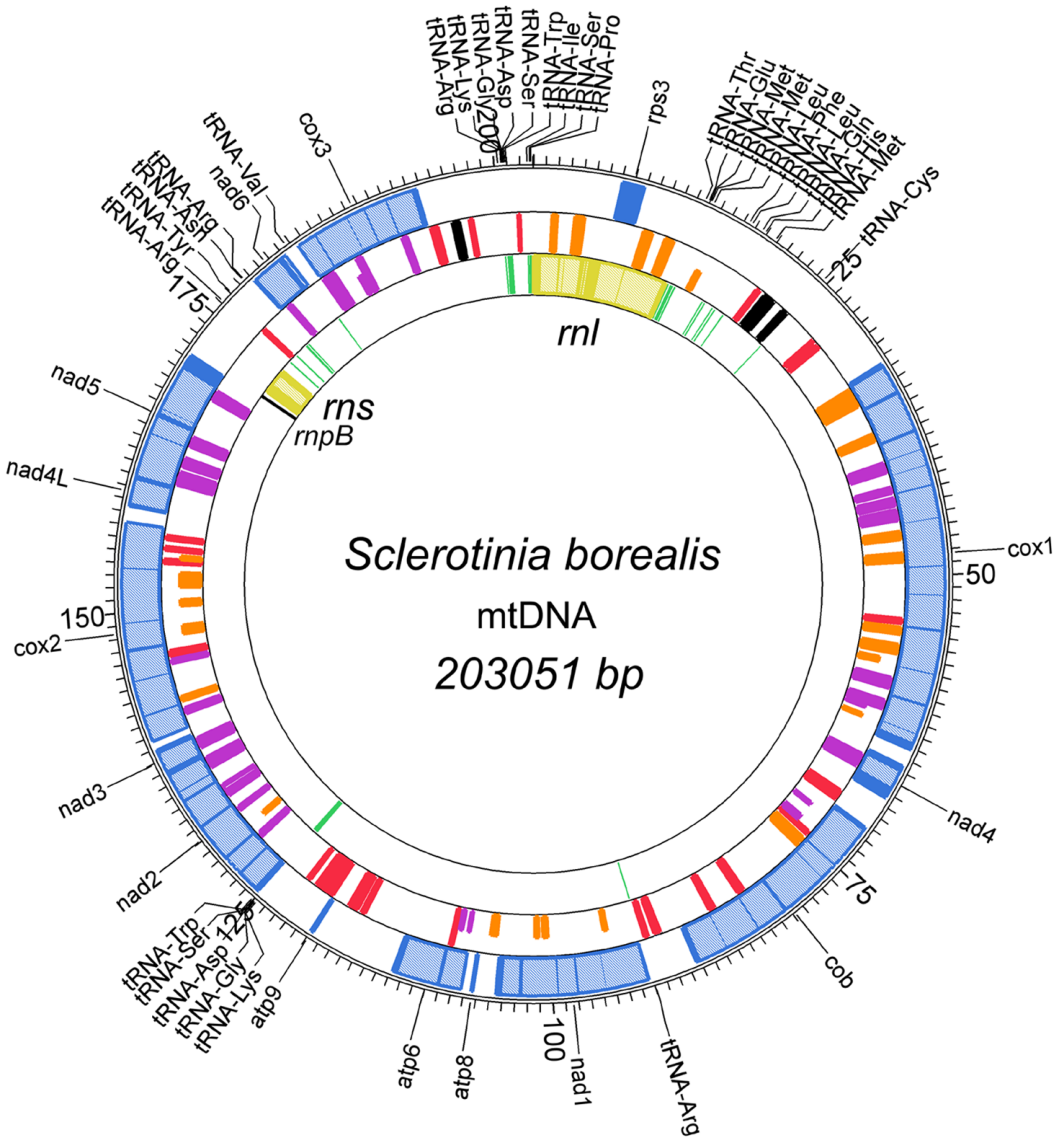
Map of the mitochondrial genome of *S. borealis*. The first ring from the outside represents the *S. borealis* core mitochondrial protein-coding genes and *rps3* (blue boxes). The second ring from the outside represents the hypothetical ORFs in red, ORFs for proteins containing GIY-YIG domains in orange, ORFs for proteins containing LAGLIDADG domains in purple, and fragments of DNA polymerase B and RNA polymerase genes in black. Full-size boxes indicate proteins containing complete GIY-YIG or LAGLIDADG domains, and half-size boxes indicate incomplete domains. The third ring represents the *rns* and *rnl* genes in yellow, *rnpB* in black and tRNA genes in green. Exons are indicated by dark colours, and introns are in light colours. All genes are oriented clockwise except for one fragment of DNA polymerase B (22818–21772 nt).

The mt genome of *S. borealis* is a circular-mapping DNA molecule of 203,051 bp with a low GC content (32.1%; Fig. 1). It contains a usual set of protein and RNA coding genes found in previously sequenced mt genomes of ascomycetes (Table 1). In addition to 31 tRNA genes and genes for the large and small ribosomal RNA (*rnl, rns*), RNA-encoding genes include a predicted *rnpB* gene encoding RNAse P (mtP-RNA), which is known to be responsible for tRNA processing [33].

**Table 1:** Genes encoding 14 typical mitochondrial proteins and the ribosomal RNA subunits in *S. borealis* mitochondrial genome.

Gene	Introns	Gene size, bp	CDS size, bp	% of intronic sequences
*cox1*	13	31646	2073	93.4
*cox2*	6	17584	756	95.7
*cox3*	5	10309	810	92.1
*cob*	6	17133	1197	93.0
*nad1*	5	12150	1092	91.0
*nad2*	6	11796	1698	85.6
*nad3*	1	1891	441	76.7
*nad4*	1	2856	1470	48.5
*nad4L*	1	2221	270	87.8
*nad5*	4	10691	1980	81.5
*nad6*	2	3137	693	77.9
*atp6*	2	5566	771	86.1
*atp8*	0	147	147	0
*atp9*	0	225	225	0
*rnl*	8	13441	2842	78.9
*rns*	1	2598	1532	41.0

Fourteen ORFs represent typical mt genes that encode subunits of the electron transport chain and of the ATP-synthase complex: seven subunits of electron transport complex I (*nad1*, *nad2*, *nad3*, *nad4*, *nad4L*, *nad5*, and *nad6*), one subunit of complex III (*cob*), three subunits of complex IV (*cox1*, *cox2*, and *cox3*), and three F0 subunits of the ATP-synthase complex (*atp6*, *atp8*, and *atp9*). In addition, there is a gene encoding the 40S ribosomal protein S3 (*rps3*), located within an intron in the *rnl*, as is the case in most of filamentous ascomycetes [34]. All of these protein-coding ORFs are transcribed in the same direction and start with the canonical translation initiation codon, AUG. The preferred stop codon was UAA, with the exception of *cox3*, *nad1*, *nad3*, and *nad6*, which used UAG. ORFs encoding typical protein-coding mt genes contain 52 introns, the *rnl* gene contains 8 introns, and one intron was found in *rns*.

In addition to typical mitochondrial genes, we also found 80 ORFs, including 61 located within introns, and 19 free-standing ORFs (Table S4). 52 intronic ORFs were predicted to encode proteins that exhibit similarities to homing endonucleases of LAGLIDADG (30 ORFs) and GIY-YIG (22 ORFs) families. 9 intronic ORFs were predicted to encode hypothetical proteins. Among free-standing ORFs, we found 2 putative homing endonuclease genes, three ORFs that are truncated fragments of DNA polymerase and RNA polymerase genes, and 14 ORFs encoding hypothetical proteins. At least 21 ORFs are probable pseudogenes, since their predicted protein products contain incomplete functional domains (Table S4).

Some fungal mitochondrial genomes contain a high fraction of repeat sequences which could account for an increase in the genome size, as observed for the mt genomes of *Agaricus bisporus* [35] and *R. solani* [7]. Dot plot analysis shows the lack of long duplicated regions in the *S. borealis* mt genome (Figure S1), and a BLASTN similarity search reveals that repeated sequences account for only about 6.6% of *S. borealis* mtDNA. Similar values were obtained for the mt genomes of *S. sclerotiorum* (6.5%), *B. fuckeliana* (4.2%) and *P. subalpina* (6.0%), suggesting that the accumulation of repeated sequences is not the primary reason for mitochondrial genome expansion in *S. borealis*.

### Transfer RNAs and genetic code

Similar to many mt genomes of *Pezizomycotina*, tRNA genes are clustered in the mtDNA of *S. borealis* (Fig. 1). The main cluster, consisting of 20 tRNAs, is located around the *rnl* gene. Two clusters including five and four tRNA genes are located between *atp9* and *nad2*, and between *rns* and *nad6*, respectively. A set of 31 tRNA genes (Table S1) is sufficient to decode all of the codons present in the predicted ORFs, except for GCN, since we did not find the corresponding tRNA gene. The presence of tRNA-W (anticodon UCA) recognising the TGA codon suggests that *S. borealis* mt protein coding genes are translated according to genetic code 4 (yeast mitochondrial genetic code).

The frequency of codon usage, summarised in Table S2, shows that all possible codons are used. AT-rich codons are much more abundant, reflecting the high AT content of the *S. borealis* mt genome. Codons for amino acids with nonpolar side chains (Phe, Leu, and Ile) are very frequent, which is not surprising given the hydrophobic nature of the proteins of respiratory membrane complexes. It is likely that abundant AUA isoleucine codons are read by one of the three predicted tRNA-M after the cytosine to lysidine modification of the CAU anticodon, such as that occurs in some plant and fungal mitochondrial genomes [36].

### Phylogenetic analysis

**Figure 2:**
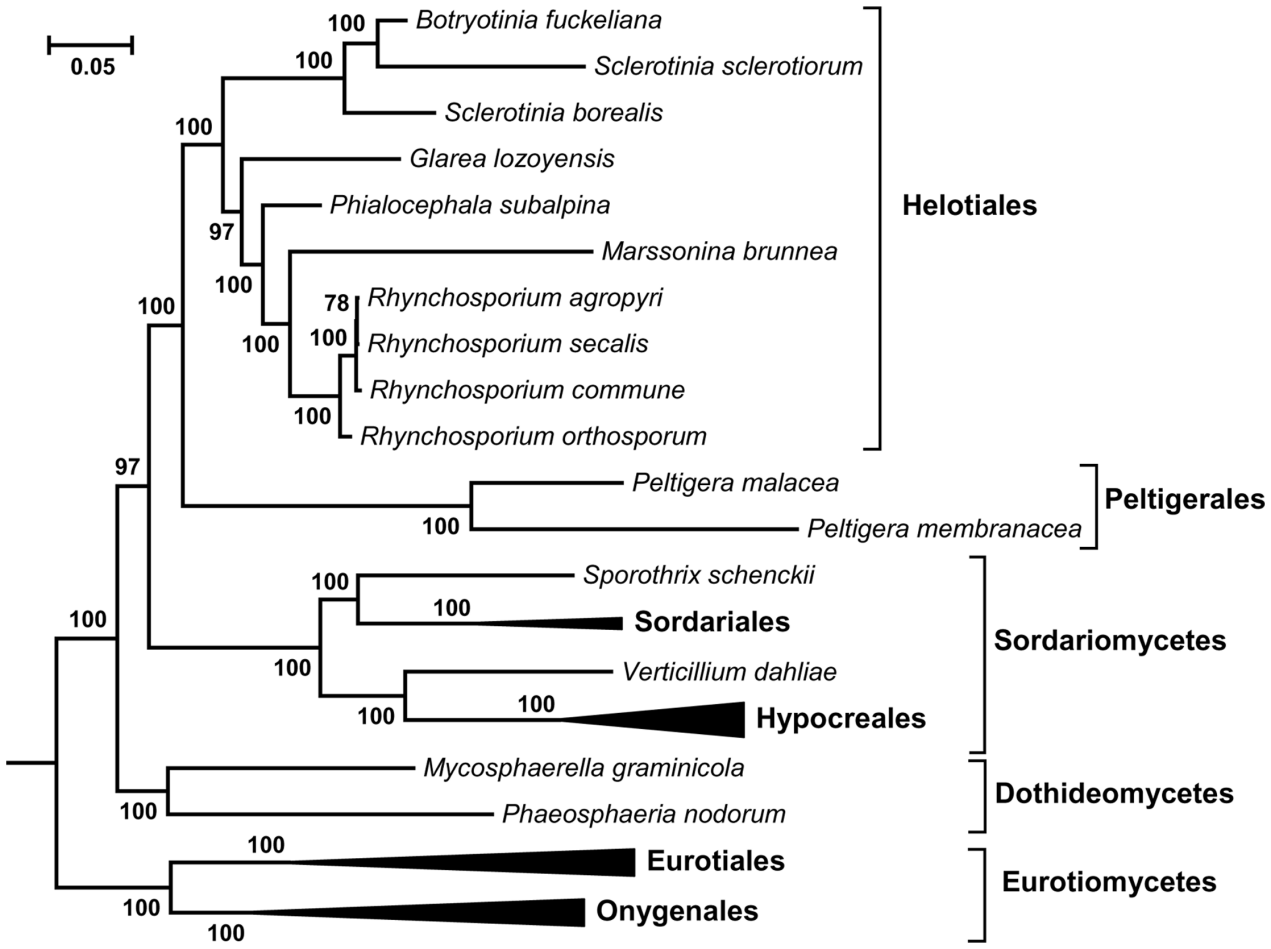
The phylogenetic tree was calculated from the multiple sequence alignment of concatenated mtDNA-encoded proteins of 51 fungal species. A dataset of 14 proteins was used, and topology was inferred using Bayesian method. Numbers above the nodes indicate bootstrap support values. The tree is drawn to scale, with branch lengths measured by the number of substitutions per site. Species analysed are shown in Table S3; only the *Ascomycota* branch of the whole tree is shown.

Previously, phylogenetic relationships among *Pezizomycotina*, a subphylum of *Ascomycota* comprising the order *Helotiales*, were established based on the comparison of five nuclear genes (*SSU rDNA, LSU rDNA*, *RPB1*, *RPB2*, and *EF-1α*) [37]. Here, we used 14 core mt proteins for the phylogenetic analysis of *Ascomycota*. Our phylogenetic analysis, performed by both Bayesian (Fig. 2) and maximum likelihood (Fig. S2) methods, produced similar trees and confirmed the affiliation of *S. borealis* with the order of *Helotiales,* together with *P. subalpina*, *G. lozoyensis*, *M. brunnea, Rhynchosporium sp., B. fuckeliana*, and *S. sclerotiorum*. *P. subalpina, G. lozoyensis*, *M. brunnea* and *Rhynchosporium sp.* appeared to form a separate branch among the analysed *Helotiales*, while *Sclerotinia* and *Botrytinia* species formed another branch and probably represent a single genus, since *S. borealis* is a sister lineage of a group comprising *B. fuckeliana* and *S. sclerotiorum*. (Fig. 2). Five classes of filamentous ascomycetes are clearly distinguished as monophylectic groups (Fig. 2 and S2): *Leotiomycetes* (represented by *Helotiales*), *Lecanoromycetes* (represented by *Peltigerales*), *Sordariomycetes*, *Dothideomycetes* and *Eurotiomycetes*. Most nodes in these trees have high bootstrap values, which indicate the robustness of the computed trees. In previous studies, *Sordariomycetes* and *Leotiomycetes* were resolved with moderate support, as they share a most recent common ancestor, with *Lecanoromycetes* as a sister group [37], [38]. The present analysis identifies *Peltigerales* as the closest relative of the *Leotiomycetes,* while *Sordariomycetes* is a more deeply branching lineage (Fig. 2). Phylogenetic analysis involving more representatives of *Lecanoromycetes* and *Leotiomycetes* would help to clarify relationships between these lineages.

### Gene order comparison

**Figure 3:**
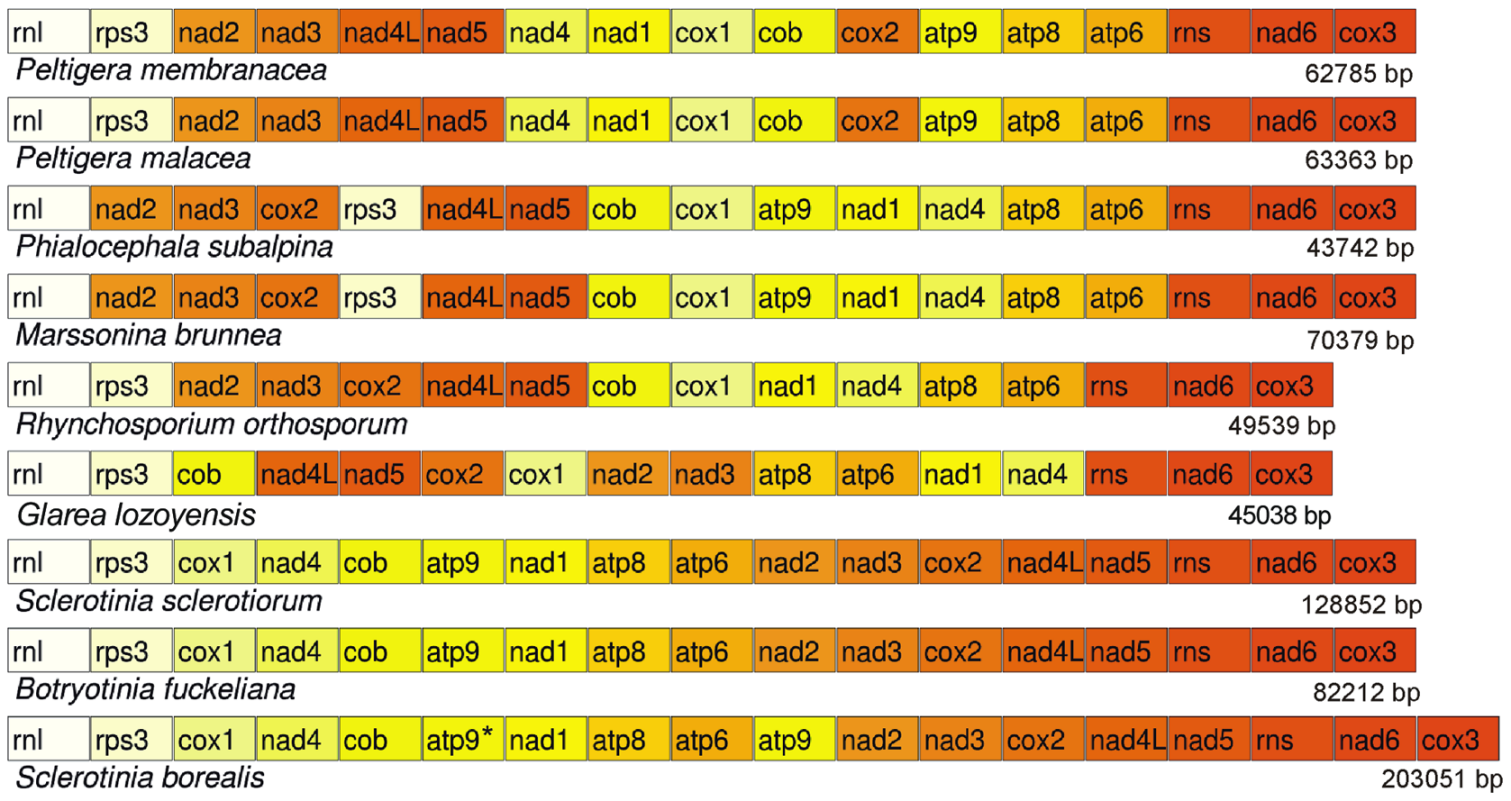
Mitochondrial genome rearrangements observed among *Helotiales* and *Peltigerales*. The second truncated copy of *atp9* in *S. borealis* mtDNA is marked by an asterisk.

Previously, the mt genomes of three helotialean species, *P. subalpina*, *S. sclerotiorum* and *B. fuckeliana,* were compared by Duo et al., 2012 [16]. We extended this analysis and included the data on the order of conserved genes in the mtDNAs of *S. borealis, G. lozoyensis*, *M. brunnea, Rhynchosporium sp.,* and two *Peltigera* species. Genome alignments (Fig. 3) reveal rearrangements between *Peltigerales* and *Helotiales* species, as well as within *Helotiale*s, between the *P. subalpina / M. brunnea / Rhynchosporium sp.* group and the *Sclerotinia*/*Botryotinia* group. The arrangement of mt genes in *G. lozoyensis* deviates significantly from both groups. In contrast, the three compared species of *Sclerotiniaceae* have almost complete synteny in gene order, with minor differences (location of *atp9*) attributed to duplication events. The gene order in the mt genomes of the second group of *Helotiales*, comprising *P. subalpina, M. brunnea* and *Rhynchosporium sp.*, is also well conserved and differs only in the presence/absence of *atp9* and location of *rps3*.

The conservation of gene order among *Sclerotinia*/*Botryotinia* species contrasts drastically with the differences in mtDNA size. *S. borealis* has the largest genome (203.1 kbp), while other species have smaller mtDNAs: 128.9 kbp in *S. sclerotiorum* and 82.2 kbp in *B. fuckeliana.*

### *S. borealis* mtDNA contains duplicated copies of some genes

**Figure 4:**
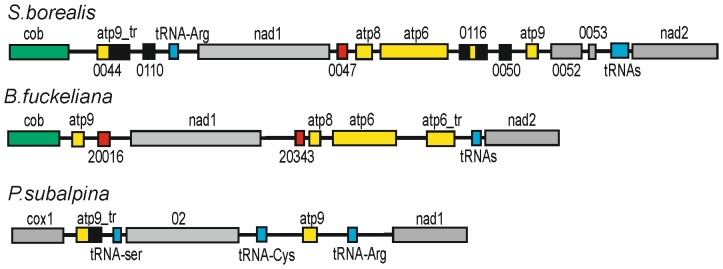
Genome organisation of the region around *atp9* genes for the helotialean species. Boxes represent ORFs and tRNA genes (blue, tRNA genes; yellow, *atp* ORFs and gene fragments; green, *cob* ORFs and gene fragments; black, inserts in truncated copies of genes; red, intronic HEG-like ORFs; grey, other ORFs). The second truncated copies of *atp6* and *atp9* are shown as atp6_tr and atp9_tr, respectively.

The large size of *S. borealis* mtDNA may be caused by duplications of some genome regions. Such events that resulted in the appearance of truncated extra copies of *atp6* and *atp9* genes were identified. The mtDNA of *S. borealis* contains two ORFs with homology to *atp9* (Fig. 4). The first ORF is located upstream of *nad2* and probably encodes an intact 74-aa long Atp9 protein. The second *atp9*-like ORF 0044 is situated between the *cob* and *nad1* genes. It consists of a full-size *atp9* sequence (96% identity over 222-bp coding sequence) lacking a stop codon, and followed by an unrelated sequence capable of encoding the 213-aa C-terminal peptide.

Analysis of the *cob*-*nad2* region of *S. borealis* and *B. fuckeliana* revealed extensive synteny between these regions (Fig. 4). However, in *B. fuckeliana,* an intact copy of *atp9* is located between *cob* and *nad1*, while the second copy between *atp6* and *nad2* is missing. Two copies of the *atp9*-related ORFs were also found in related species, including *P. subalpina* [16], thus indicating that gene duplication and the subsequent truncation of one of the two copies of *atp9* is a common feature of mt genomes of helotialean species.

In another case, duplication of relatively short piece of gene was predicted. The region between *nad1* and *nad2* also contains two ORFs showing sequence similarity to *atp6*. The first is an intact full-size *atp6*, while the second downstream 384-bp long ORF 0116 contains an internal 183-bp fragment of *atp6* (80% nucleotide sequence identity). Such an arrangement may have resulted from a tandem duplication event. An additional truncated copy of the *atp6* gene is also present in *B. fuckeliana* mtDNA (Fig. 4).

### Introns in *S. borealis* mtDNA

Sequences encoding 14 typical mitochondrial proteins and the ribosomal RNA subunits (*rnl* and *rns*) totalled 18 kb and represent only 9% of the entire genome. The 61 introns (Table 1) identified in core mitochondrial protein-coding genes and the *rns* and *rnl* genes account for 125 kb (62% of the genome). The introns range in size from 251 nt for intron 1 in the *rnl* gene, to 4300 nt for intron 5 in the *cox2* gene. These introns are scattered throughout the genome (Fig. 1 and Table 1) and are found in 12 of the 15 protein-coding genes (52 introns), in the *rnl* gene (8 introns) and in the *rns* (1 intron). Specifically, 13 introns were found in the *cox1* gene, accounting for 93.4% of the gene sequence. The *cob*, *nad2*, and *cox2* genes have six introns each. The *nad1*, *cox3*, and *nad5* genes possess four introns each. The *nad6* and *atp6* genes have two introns each, while the *nad4*, *nad4L*, and *nad3* contain only a single intron each.

35 ORFs related to homing endonucleases (HEG) [39] of the LAGLIDADG (23 ORFs) and GIY-YIG families (12 ORFs) were found in introns. In addition, 17 HEG-related intronic HEG-related ORFs are probably pseudogenes, since their predicted products contain incomplete LAGLIDADG (7 ORFs) or GIY-YIG (10 ORFs) domains (Table S4). Two HEG-related ORFs containing a partial GIY-YIG domain were identified outside of core mitochondrial genes (Table S4). These free-standing HEG-related ORFs have limited similarity (35–53% amino acid sequence identity) to intronic ORFs of phylogenetically remote fungal species, but no significant similarity to any *S. borealis* proteins (Table S4), which suggests their acquisition via lateral gene transfer rather than by intragenomic proliferation.

We compared the locations of introns in the mt genomes of *S. borealis* and the phylogenetically related organisms *B. fuckeliana*, *Rhynchosporium commune, Rhynchosporium orthosporum*, *P. malacea*, and *P. membranacea* (Table 2). *S. sclerotiorum* was not included in this analysis since its mitochondrial genome was not annotated and intron locations are unknown. Comparative analysis showed that most introns are present in two genes: *cox1* and *cob* (Table 2). Many orthologous introns are located in the same positions in all of the analysed species.

**Table 2:** Location of introns in the mtDNAs of Helotiales and Peltigerales species.

Gene	Position of intron*	Presence/absence of orthologous introns					
		Sbor	Bfuc	Pmal	Pmem	Rcom	Rorth
*cob*	**63**	**-**	**+ (H)**	**-**	**-**	**-**	**-**
	**93**	**+ (eH)**	**-**	**-**	**-**	**-**	**-**
	**131**	**+ (H)**	**+ (H)**	**+ (eH)**	**+ (H)**	**-**	**-**
	**145**	**+**	**+ (H)**	**-**	**-**	**-**	**-**
	**164**	**-**	**+ (eH)**	**+**	**+**	**-**	**-**
	**227**	**+**	**-**	**-**	**-**	**-**	**-**
	**260**	**+**	**-**	**-**	**-**	**-**	**-**
	**270**	**+**	**-**	**-**	**-**	**-**	**-**
	**274**	**-**	**-**	**+ (eH)**	**+**	**-**	**-**
*cox1*	**107 (D)**	**+ (H)**	**+ (H)**	**+ (H)**	**+ (H)**	**-**	**-**
	**117 (G)**	**-**	**-**	**+ (H)**	**-**	**-**	**-**
	**130 (I)**	**-**	**-**	**-**	**-**	**+**	**-**
	**166 (K)**	**-**	**+ (H)**	**+ (H)**	**+ (H)**	**+ (H)**	**+ (H)**
	**201 (L)**	**+ (H)**	**+ (H)**	**-**	**-**	**-**	**-**
	**241(N)**	**-**	**-**	**-**	**-**	**+**	**-**
	**273 (P)**	**+**	**+ (H)**	**+ (H)**	**+ (H)**	**-**	**-**
	**277 (Q)**	**+ (H)**	**-**	**-**	**-**	**+ (H)**	**-**
	**280 (S)**	**+ (H)**	**-**	**-**	**-**	**+ (H)**	**-**
	**306 (T)**	**+ (H)**	**-**	**-**	**-**	**-**	**-**
	**310 (U)**	**+ (H)**	**-**	**-**	**-**	**+**	**-**
	**326 (W)**	**+**	**+**	**+ (H)**	**+ (H)**	**+ (H)**	**-**
	**337 (Y)**	**+**		**+ (H)**	**-**	**+ (H)**	**-**
	**389 (AC)**	**+ (H, eH)**	**+ (H)**	**-**	**-**	**-**	**-**
	**407 (AD)**	**+ (H)**	**-**	**-**	**+ (H)**	**+ (H)**	**-**
	**414 (AE)**	**-**	**-**	**+ (eH)**	**-**	**-**	**-**
	**449 (AF)**	**-**	**-**	**-**	**-**	**+ (eH)**	**-**
	**459 (AG)**	**-**	**+ (H)**	**-**	**-**	**+ (H)**	**-**
	**469 (AH)**	**+ (H, eH)**	**+ (H)**	**+ (eH)**	**+ (H)**	**+**	**+**
	**472 (AI)**	**+ (eH)**	**-**	**-**	**-**	**-**	**-**
*cox2*	**36**	**+ (H)**	**-**	**-**	**-**	**-**	**-**
	**76**	**+ (H)**	**-**	**-**	**-**	**+ (H)**	**-**
	**106**	**+ (H)**	**-**	**-**	**-**	**-**	**-**
	**116**	**-**	**-**	**-**	**-**	**+ (H)**	**-**
	**181**	**+ (eH)**	**-**	**-**	**-**	**-**	**-**
	**197**	**+ (eH)**	**-**	**+**	**-**	**-**	**-**
	**216**	**+ (eH)**	**+ (H)**	**-**	**-**	**+ (H)**	**-**
*cox3*	**36**	**+ (H)**	**-**	**-**	**-**	**-**	**-**
	**73**	**+ (H, eH)**	**+ (H)**	**-**	**-**	**-**	**-**
	**80**	**+**	**-**	**-**	**-**	**-**	**-**
	**111**	**+**	**-**	**-**	**-**	**-**	**-**
	**142**	**+ (H)**	**-**	**-**	**-**	**-**	**-**
	**185**	**-**	**+ (H)**	**-**	**-**	**-**	**-**
	**215**	**-**	**+ (H)**	**+**	**-**	**-**	**-**
*nad1*	**48**	**+ (eH)**	**-**	**-**	**-**	**-**	**-**
	**55**	**+**	**-**	**-**	**-**	**-**	**-**
	**97**	**+**	**-**	**-**	**-**	**-**	**-**
	**129**	**+ (eH)**	**+ (H)**	**-**	**-**	**-**	**-**
	**212**	**+**	**-**	**-**	**-**	**-**	**-**
*nad2*	**128**	**+**	**+ (H)**	**-**	**-**	**-**	**-**
	**185**	**+ (H)**	**-**	**-**	**-**	**-**	**-**
	**268**	**+ (H, eH)**	**-**	**-**	**-**	**-**	**-**
	**342**	**+ (H, eH)**	**-**	**-**	**-**	**-**	**-**
	**412**	**+**	**-**	**-**	**-**	**-**	**-**
	**442**	**+ (H)**	**-**	**-**	**-**	**-**	**-**
*nad3*	**29**	**+ (H)**	**-**	**-**	**-**	**-**	**-**
*nad4*	**170**	**+ (H)**	**-**	**-**	**-**	**-**	**-**
*nad4L*	**80**	**+**	**+**	**+ (eH)**	**+ (eH)**	**-**	**-**
*nad5*	**82**	**+ (H)**	**-**	**-**	**-**	**-**	**-**
	**108**	**+ (H)**	**-**	**-**	**-**	**-**	**-**
	**142**	**-**	**-**	**-**	**+ (H)**	**-**	**-**
	**236**	**+**	**-**	**-**	**+**	**-**	**-**
	**239**	**+ (H)**	**-**	**-**	**+ (eH)**	**-**	**-**
	**253**	**-**	**-**	**-**	**-**	**+ (H)**	**-**
	**310**	**-**	**-**	**+ (eH)**	**-**	**+ (eH)**	**-**
*nad6*	**78**	**+ (H)**	**-**	**-**	**-**	**-**	**-**
	**229**	**+**	**-**	**-**	**-**	**-**	**-**
*atp6*	**24**	**+ (eH)**	**-**	**-**	**-**	**-**	**-**
	**113**	**+**	**+ (H)**	**-**	**-**	**-**	**-**
Total **		**52**	**20**	**15**	**13**	**17**	**2**

Plus (+) and minus (−) symbols indicate, respectively, presence or absence of orthologues intron in particular position. H and eH in parentheses represents the presence of the putative functional and eroded HEG-like ORFs, respectively.

Sbor – *S. borealis* F-4128, Bfuc - *B. fuckeliana* B05.10, Pmal – *P. malacea* DB3992, Pmem – *P. membranacea* LA-31632, Rcom – *R. commune* UK7, Rorth - *R. orthosporum* 04CH-BAR-A.1.1.3.

*The intron positions are indicated relative to the amino acid sequences of reference intronless genes of *P. subalpina* UAMH 11012. For *cox1* introns the nomenclature of insertion sites suggested by Ferandon et al (2010) is shown in parentheses.

**Total number of introns in analysed genes. Note the absences of introns in the *atp8* and *atp9* genes in all analysed species.

The *cox1* gene is the most common target for insertions of group I introns. Introns in this gene were found in different taxonomic groups, and the position of insertion may be used for analysis of intron mobility during evolution [26]. Among the 13 introns found in the *cox1* gene in *S. borealis*, eight introns (1, 2, 3, 4, 6, 8, 9, 10) have a high sequence identity to orthologous introns found in the same positions in *cox1* genes of other *Helotiales* and *Peltigerales* (Table S5), suggesting a common origin. For example, the nucleotide sequence of intron 3 of the *S. borealis cox1* is 95% identical to the corresponding *cox1* intron in *B. fuckeliana* (Table S5). Three introns (5, 11, 13) have a higher sequence similarity to introns of other fungi (Table S5), suggesting their acquisition through horizontal transfer.

Overall, only 18 introns found in protein-coding genes showed the highest nucleotide sequences similarity to mt introns of *Helotiales* (17 introns) and *Peltigerales* (1 introns), while 29 introns are most similar to introns of other fungal lineages, in some instances even distantly related (e.g., intron 6 of *nad2* is homologous to the mtDNA intron of the oomycete *Albugo laibachii*). Two *cox1* introns (7 and 12) and three introns located in other protein-coding genes (*cob* intron 4, *nad1* intron 4 and *nad6* intron 2) show no homology to other fungal introns (Table S5), and also may originate from phylogenetically remote fungal species.

Among 9 introns found in RNA-coding genes, only three are most similar to rRNA introns of *Helotiales*, while four share the highest identity with introns of mtDNA of other fungi, and two introns did not show significant similarity to sequences deposited in GenBank.

**Figure 5:**
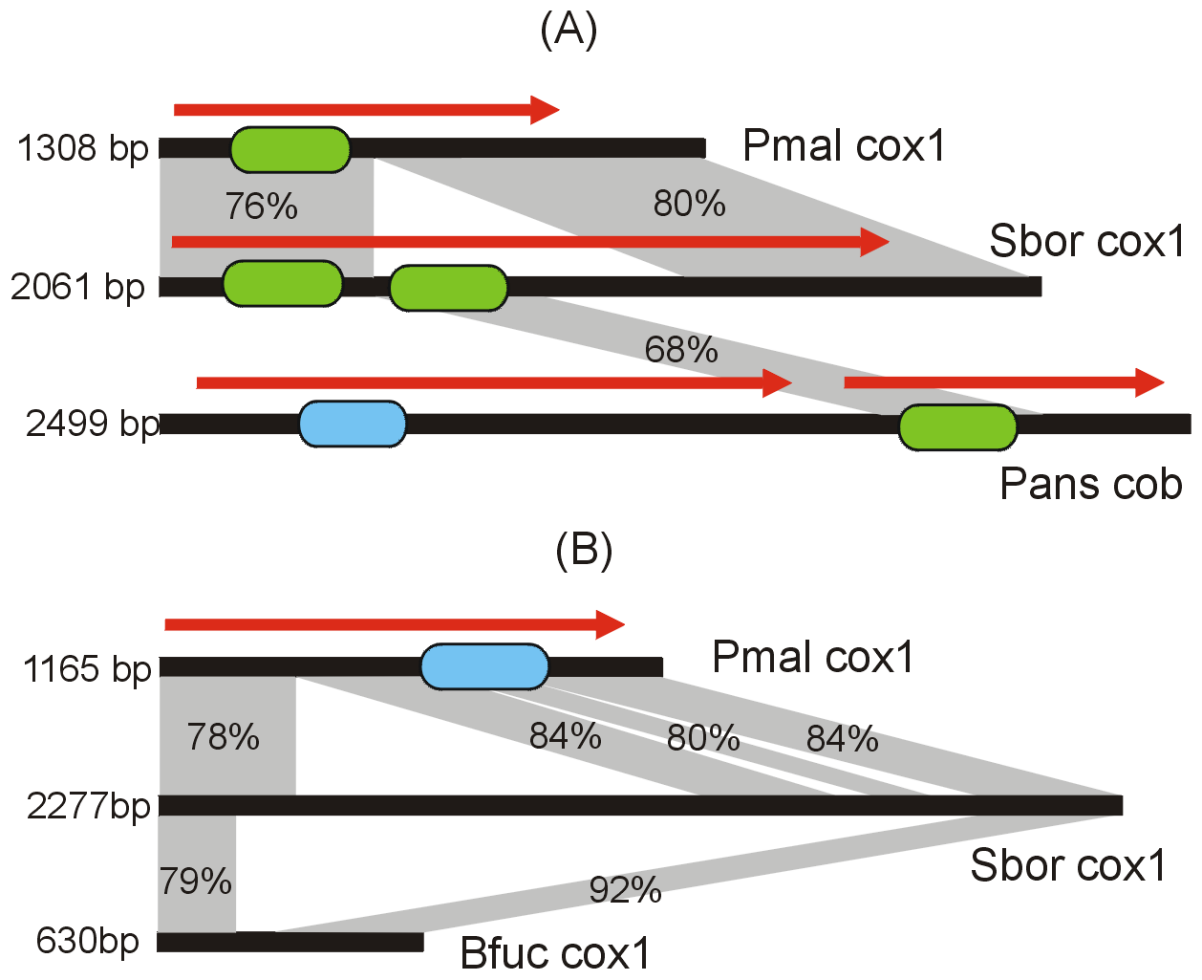
Structure of the *S. borealis cox1* gene introns 1 (A) and 8 (B). Introns are represented by black horizontal bars; the sizes are drawn to scale. Red arrows show HEG-like ORFs. Green and blue rectangles show, respectively, catalytic GIY-YIG_bI1_like (cd10445) and LAGLIDADG_1 (pfam00961) domains. Nucleotide sequence similarity is indicated by grey areas between introns. Sbor – *S. borealis*, Bfuc – *B. fuckeliana*, Pmal – *P. malacea*, Pans – *P. anserina*.

Analysis of nucleotide sequences of the orthologous introns in the *cox1* gene reveal events of multiple insertions during intron evolution (Fig. 5A). The first 2061 bp long intron has two regions with 76–80% nucleotide sequence identity with the 1308 bp intron present at the same position in the *cox1* genes of *P. malacea* [40]. The central region (about bp 500–1250) of this intron in *S. borealis* is similar to an internal region of a 2499 bp long intron of the *cob* gene of phylogenetically distant fungus, *Podospora anserina* of the order *Sordariales*, suggesting the additional insertion of a new sequence into an already present intron. This insertion occurred in-frame within the HEG-like ORF present in the first intron, thus resulting in the appearance of a second GIY-YIG catalytic domain in the intron-encoded protein (Fig 5A). The closest relative of *S. borealis*, the helotialean fungus *B. fuckeliana*, harbours in this position an intron with a dissimilar nucleotide sequence. It can be assumed that insertion of the intron occurred in a common ancestor of *Peltigerales* and *Helotiales*, and it was then replaced by another intron in *B. fuckeliana*, while an extra insertion occurred in *S. borealis* (Fig5A).

Three insertions disrupting the HEG-like ORF occur in intron 8 of *cox1* in *S. borealis* (Fig 5B). Excluding these insertions, the nucleotide sequence of this intron is about 80% identical to the corresponding introns present at the same positions in *P. malacea*. An orthologous but shorter intron is present at this position in *B. fuckeliana*; it may result from internal deletion within the parental intron (Fig 5B).

Overall, our analysis reveals possible evolutionary events, including insertions of introns and the horizontal transfer of introns from remote fungal species, that have led to an increase in the size of the *S. borealis* mt genome.

### Plasmid-like sequences integrated in the mtDNA

In addition to the introns, plasmids represent other types of mobile genetic elements often found in the mitochondria of fungi and plants [41]. These plasmids can be linear or integrated into the mt genome [42]. Most contain two ORFs, one encoding a family B DNA polymerase, and the second encoding a DNA-dependent RNA polymerase subunit, as well as several other ORFs [42]. The majority of mt plasmids have been predicted based on the analysis of genomic data [43], [44]. It has been shown that such plasmids can be integrated into the mt genomes [43], [45], [46], [47], [48], [49]. Upon integration, they accumulate mutations and deletions, and are finally eliminated during evolution. A region in the *S. borealis* mtDNA located between the *rnl* and *cox1* genes contains an ORF 0018 encoding a 1047 bp fragment of DNA polymerase B, and a nearby located 540 bp fragment of the RNA polymerase gene (ORF 0019). The second region, located downstream of the *cox3* gene, contains a 591 bp long ORF 0093 for the DNA polymerase B fragment. It can be assumed that these regions may have resulted from an ancient integration of plasmids, which were then mostly eliminated from the mtDNA of *S. borealis*. The sequences of the two DNA polymerase B genes have a low homology (22% amino acid identity), suggesting the different origin of these two insertions.

### Conclusions

Our data on the organisation of the mt genome of *S. borealis,* and its comparison with mtDNA sequences of related species, reveal its specific features and the general characteristics of mtDNAs of the order *Helotiales* [16], [40]. Our comparative analysis indicates that the 203,051 bp long *S. borealis* mt genome is the second largest fungal mt genome sequenced to date, after the 235,849 bp long mtDNA of the basidiomycete *R. solani* Rhs1AP [7]. The next largest mitochondrial genomes are the *R. solani* AG1-IB (162,751 bp) [50] and *A. bisporus* (135,005 bp) [35]. Expansion of the mitochondrial genome size in *R. solani* Rhs1AP was driven by accumulation of introns, HEG-like genes and hypothetical genes, but the most peculiar feature was the presence of various interspersed repeats, which occupy about one third of the genome [7].

In contrast, the major contributors to the large size of the *S. borealis* mt genome are introns, while different repeats accounted for less than 7% of the genome sequence. The number of introns found in *S. borealis* (61) is higher than in the mt genomes of *R. solani* Rhs1AP (43) and *A. bisporus* (46). Introns identified in the core mitochondrial genes accounted for 125 kb. The actual contribution of intron-related sequences to the genome size is probably higher, since their insertion in intergenic regions, evidenced by the finding of several free-standing HEG-related ORFs, will increase their proportion in the mtDNA. The mt genome of *S. borealis* contains a large number of introns found in different lineages of fungi, including phylogenetically distant ones, thus indicating the important role of the horizontal transfer of introns in the evolution of mt genomes. Extension of intergenic regions, accounting for about 50 kb (23% of the genome), is another major contributor to the large size of the *S. borealis* mt genome. Duplications of some regions and insertion of plasmids also increase the size of the genome.

Interestingly, phylogenetically related fungi of the orders *Peltigetales* and *Helotiales* differ in mtDNA length and the number of introns. A notable feature is the complete absence of introns in the mt genome of *P. subalpina* [16]. To the contrary, many introns found in *S. borealis* are also present in the mtDNAs of the representatives from the more deeply branching order *Peltigerales* (*P. malaceae* and *P. membranaceae*) (Table S5). These observations imply a dynamic pattern of intron acquisition and loss during evolution: in some lineages, introns were completely lost, while in others, extensive accumulation of introns occurred. It is likely that the *S. borealis* genome has efficient mechanisms for intron acquisition and retention, but the nature of these mechanisms remains unknown.

## Supporting Information

Figure S1 **Dot plot analysis of** ***S. borealis*** **mtDNA performed with Dotmatcher**. (http://emboss.bioinformatics.nl/cgi-bin/emboss/dotmatcher). The main diagonal represents the sequence's alignment with itself; lines off the main diagonal represent repetitive patterns within the sequence. Each dot represents a 100 bp significantly matching segment.
(PDF)

Figure S2 **The phylogenetic tree was calculated from the multiple sequence alignment of 14 concatenated mtDNA-encoded proteins.** Topology was inferred using Maximum-Likelhood method. Numbers above the nodes indicate bootstrap support values. The tree is drawn to scale, with branch lengths measured by the number of substitutions per site. Species analyzed are shown in the Table S3, only Ascomycota branch of the whole tree is shown.
(PDF)

Table S1 **The codon-anticodon recognition pattern and tRNA genes identified in** ***S. borealis*** **mitochondrial genome.**
(DOC)

Table S2 **Codon usage of protein-coding genes in** ***S. borealis*** **mitochondrial genome.**
(DOC)

Table S3 **List of mt genomes used for phylogenetic studies.**
(DOC)

Table S4 **List of ORFs identified in** ***S. borealis*** **mitochondrial genome.**
(XLS)

Table S5 **Sequence similarity betwen mt introns of** ***S. borealis*** **and introns of other organisms.**
(XLS)
