# Supplementary material for: The 203 kbp Mitochondrial Genome of the Phytopathogenic Fungus Sclerotinia borealis Reveals Multiple Invasions of Introns and Genomic Duplications
Source: PLoS One. 2014 Sep 12;9(9):e107536. doi: 10.1371/journal.pone.0107536 (PMC4162613; doi:10.1371/journal.pone.0107536)
Supplement: Figure S1 — Dot plot analysis of S. borealis mtDNA performed with Dotmatcher. (http://emboss.bioinformatics.nl/cgi-bin/emboss/dotmatcher). The main diagonal represents the sequence's alignment with itself; lines off the main diagonal represent repetitive patterns within the sequence. Each dot represents a 100 bp significantly matching segment. (PDF) [file pone.0107536.s001.pdf]

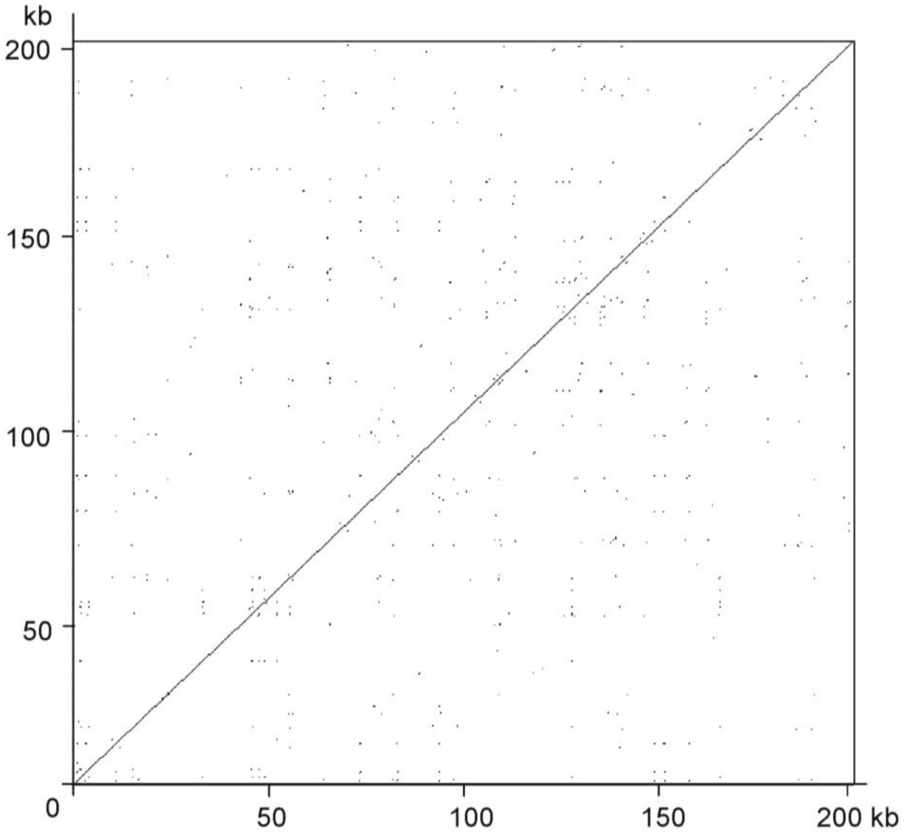

Figure S1. Dot plot analysis of *S. borealis* mtDNA performed with Dotmatcher (http://emboss.bioinformatics.nl/cgi-bin/emboss/dotmatcher). The main diagonal represents the sequence's alignment with itself; lines off the main diagonal represent repetitive patterns within the sequence. Each dot represents a 100 bp significantly matching segment.
