# Supplementary material for: The 203 kbp Mitochondrial Genome of the Phytopathogenic Fungus Sclerotinia borealis Reveals Multiple Invasions of Introns and Genomic Duplications
Source: PLoS One. 2014 Sep 12;9(9):e107536. doi: 10.1371/journal.pone.0107536 (PMC4162613; doi:10.1371/journal.pone.0107536)
Supplement: Figure S2 — The phylogenetic tree was calculated from the multiple sequence alignment of 14 concatenated mtDNA-encoded proteins. Topology was inferred using Maximum-Likelhood method. Numbers above the nodes indicate bootstrap support values. The tree is drawn to scale, with branch lengths measured by the number of substitutions per site. Species analyzed are shown in the Table S3, only Ascomycota branch of the whole tree is shown. (PDF) [file pone.0107536.s002.pdf]

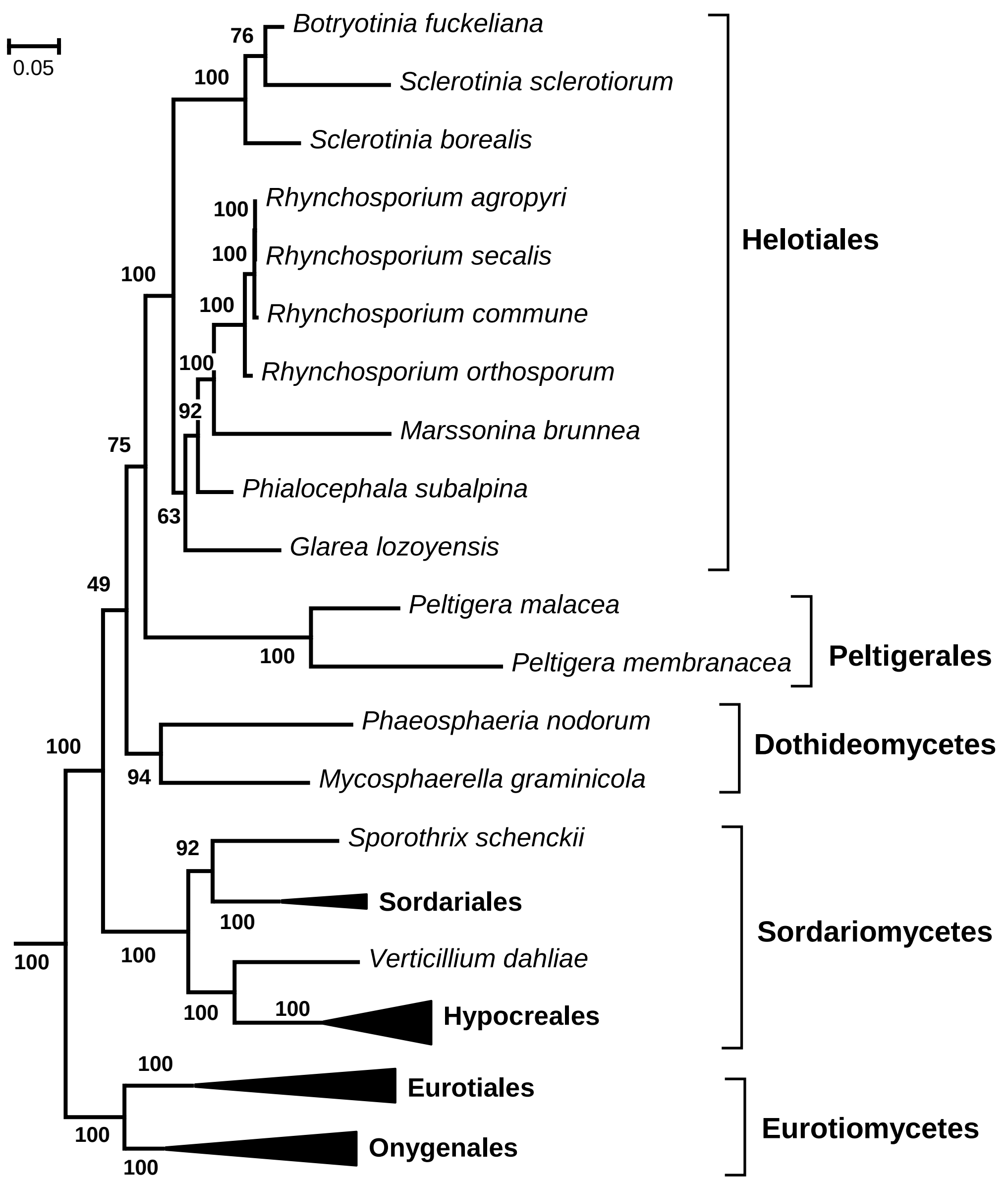

Figure S2. The phylogenetic tree was calculated from the multiple sequence alignment of 14 concatenated mtDNA-encoded proteins. Topology was inferred using Maximum-Likelihood method. Numbers above the nodes indicate bootstrap support values. The tree is drawn to scale, with branch lengths measured by the number of substitutions per site. Species analyzed are shown in the Table S3, only Ascomycota branch of the whole tree is shown.
