# Supplementary material for: The 203 kbp Mitochondrial Genome of the Phytopathogenic Fungus Sclerotinia borealis Reveals Multiple Invasions of Introns and Genomic Duplications
Source: PLoS One. 2014 Sep 12;9(9):e107536. doi: 10.1371/journal.pone.0107536 (PMC4162613; doi:10.1371/journal.pone.0107536)
Supplement: Table S1 — The codon-anticodon recognition pattern and tRNA genes identified in S. borealis mitochondrial genome. (DOC) [file pone.0107536.s003.doc]

Table S1. The codon-anticodon recognition pattern and tRNA genes identified in *S. borealis* mitochondrial genome.

| UUU | Phe | *trnF-GAA* | UCU | Ser | *trnS-TGA* | UAU | Tyr | *trnY-GTA* | UGU | Cys | *trnC-GCA* |
| --- | --- | --- | --- | --- | --- | --- | --- | --- | --- | --- | --- |
| UUC | Phe |  | UCC | Ser |  | UAC | Tyr |  | UGC | Cys |  |
| UUA | Leu | *trnL-TAA* | UCA | Ser |  | UAA | - |  | UGA | Trp | *trnW-TCA* |
| UUG | Leu |  | UCG | Ser |  | UAG | - |  | UGG | Trp | *trnW-CCA* |
|  |  |  |  |  |  |  |  |  |  |  |  |
| CUU | Leu | *trnL-TAG* | CCU | Pro | *trnP-TGG* | CAU | His | *trnH-GTG* | CGU | Arg | *trnR-TCG(2)* |
| CUC | Leu |  | CCC | Pro |  | CAC | His |  | CGC | Arg |  |
| CUA | Leu |  | CCA | Pro |  | CAA | Gln | *trnQ-TTG* | CGA | Arg |  |
| CUG | Leu |  | CCG | Pro |  | CAG | Gln |  | CGG | Arg |  |
|  |  |  |  |  |  |  |  |  |  |  |  |
| AUU | Ile | *trnI-GAT* | ACU | Thr | *trnT-TGT* | AAU | Asn | *trnN-GTT* | AGU | Ser | *trnS-GCT(2)* |
| AUC | Ile |  | ACC | Thr |  | AAC | Asn |  | AGC | Ser |  |
| AUA | Ile |  | ACA | Thr |  | AAA | Lys | *trnK-TTT(2)* | AGA | Arg | *trnR-TCT*  *trnR-CCT* |
| AUG | Met | *trnM-CAT(3)* | ACG | Thr |  | AAG | Lys |  | AGG | Arg |  |
|  |  |  |  |  |  |  |  |  |  |  |  |
| GUU | Val | *trnV-TAC* | GCU | Ala |  | GAU | Asp | *trnD-GTC(2)* | GGU | Gly | *trnG-TCC(2)* |
| GUC | Val |  | GCC | Ala |  | GAC | Asp |  | GGC | Gly |  |
| GUA | Val |  | GCA | Ala |  | GAA | Glu | *trnE-TTC* | GGA | Gly |  |
| GUG | Val |  | GCG | Ala |  | GAG | Glu |  | GGG | Gly |  |

The number shown in parentheses indicates the number of tRNA genes identified.
