# Supplementary material for: The 203 kbp Mitochondrial Genome of the Phytopathogenic Fungus Sclerotinia borealis Reveals Multiple Invasions of Introns and Genomic Duplications
Source: PLoS One. 2014 Sep 12;9(9):e107536. doi: 10.1371/journal.pone.0107536 (PMC4162613; doi:10.1371/journal.pone.0107536)
Supplement: Table S2 — Codon usage of protein-coding genes in S. borealis mitochondrial genome. (DOC) [file pone.0107536.s004.doc]

Table S2. Codon usage of protein-coding genes in *S. borealis* mitochondrial genome.

| Codon | Aa | Number | Codon | Aa | Number | Codon | Aa | Number | Codon | Aa | Number |
| --- | --- | --- | --- | --- | --- | --- | --- | --- | --- | --- | --- |
| UUU  UUC  UUA  UUG | Phe  Phe  Leu  Leu | 307  124  510  72 | UCU  UCC  UCA  UCG | Ser  Ser  Ser  Ser | 130  29  73  24 | UAU  UAC  UAA  UAG | Tyr  Tyr  -  - | 159  80  12  4 | UGU  UGC  UGA  UGG | Cys  Cys  Trp  Trp | 35  5  54  13 |
| CUU  CUC  CUA  CUG | Leu  Leu  Leu  Leu | 94  12  84  28 | CCU  CCC  CCA  CCG | Pro  Pro  Pro  Pro | 115  28  39  19 | CAU  CAC  CAA  CAG | His  His  Gln  Gln | 79  33  84  27 | CGU  CGC  CGA  CGG | Arg  Arg  Arg  Arg | 19  1  5  4 |
| AUU  AUC  AUA  AUG | Ile  Ile  Ile  Met | 193  67  266  127 | ACU  ACC  ACA  ACG | Thr  Thr  Thr  Thr | 119  14  120  23 | AAU  AAC  AAA  AAG | Asn  Asn  Lys  Lys | 167  71  164  37 | AGU  AGC  AGA  AGG | Ser  Ser  Arg  Arg | 184  45  99  15 |
| GUU  GUC  GUA  GUG | Val  Val  Val  Val | 177  23  124  51 | GCU  GCC  GCA  GCG | Ala  Ala  Ala  Ala | 180  47  83  36 | GAU  GAC  GAA  GAG | Asp  Asp  Glu  Glu | 106  35  94  48 | GGU  GGC  GGA  GGG | Gly  Gly  Gly  Gly | 196  14  74  40 |
|  |  |  |  |  |  |  |  |  |  |  |  |
