# Supplementary material for: The 203 kbp Mitochondrial Genome of the Phytopathogenic Fungus Sclerotinia borealis Reveals Multiple Invasions of Introns and Genomic Duplications
Source: PLoS One. 2014 Sep 12;9(9):e107536. doi: 10.1371/journal.pone.0107536 (PMC4162613; doi:10.1371/journal.pone.0107536)
Supplement: Table S3 — List of mt genomes used for phylogenetic studies. (DOC) [file pone.0107536.s005.doc]

Table S3. List of mt genomes used for phylogenetic studies

| Order | Name | Source of sequences |
| --- | --- | --- |
| Ascomycota | | |
| Onygenales | *Arthroderma obtusum* ATCC 42129 | NC_012830 |
|  | *Arthroderma uncinatum* ATCC 28454 | NC_012828 |
|  | *Epidermophyton floccosum* IMBST95031 | NC_007394 |
|  | *Microsporum canis* ATCC 36299 | NC_012832 |
|  | *Paracoccidioides brasiliensis* Pb18 | NC_007935 |
|  | *Trichophyton mentagrophytes* BMU 03104 | NC_012826 |
|  | *Trichophyton rubrum* BMU 01672 | NC_012824 |
| Eurotiales | *Aspergillus fumigatus* A1163 | NC_017016 |
|  | *Aspergillus nidulans* FGSC A4 | NC_017896 |
|  | *Aspergillus niger* N909 | NC_007445 |
|  | *Aspergillus tubingensis* 932 | NC_007597 |
|  | *Penicillium digitatum* pd01 | NC_015080 |
|  | *Penicillium solitum* 20-01 | NC_016187 |
|  | *Talaromyces marneffei* MP1 | NC_005256 |
| Hypocreales | *Cordyceps bassiana* Bb147 | NC_017842 |
|  | *Cordyceps brongniartii* IMBST95031 | NC_011194 |
|  | *Beauveria bassiana* Bb13 | NC_010652 |
|  | *Fusarium oxysporum* F11 | NC_017930 |
|  | *Fusarium solani* mpVI | NC_016680 |
|  | *Gibberella moniliformis* 7600 | NC_016687 |
|  | Gibberella zeae | NC_009493 |
|  | *Hypocrea jecorina* | NC_003388 |
|  | *Lecanicillium muscarium* C42 | NC_004514 |
|  | *Metarhizium anisopliae* ME1 | NC_008068 |
| Helotiales | *Botryotinia fuckeliana* B05.10 | KC832409 |
|  | *Glarea lozoyensis* 74030 | KF169905 |
|  | *Marssonina brunnea* f. sp. 'multigermtubi' | NC_015991 |
|  | *Phialocephala subalpina* 70-1 | NC_015789 |
|  | *Rhynchosporium commune* UK7 | NC_023126 |
|  | *Rhynchosporium secalis* 02CH4-6a.1 | NC_023128 |
|  | *Rhynchosporium agropyri* 04CH-RACA.6.1 | NC_023125 |
|  | *Rhynchosporium orthosporum* 04CH-BAR-A.1.1.3 | NC_023127 |
|  | *Sclerotinia sclerotiorum* 1980 UF-70 | contig derived from [http://www.broadinstitute.org](http://www.broadinstitute.org/) |
| Peltigerales | *Peltigera malacea* DB3992 | NC_016955 |
|  | *Peltigera membranacea* LA-31632 | NC_016957 |
| Ophiostomatales | *Sporothrix schenckii* ATCC 10268 | NC_015923 |
| Sordariales | *Chaetomium thermophilum* var. thermophilum DSM 1495 | NC_015893 |
|  | Madurella mycetomatis | NC_018359 |
|  | *Podospora anserina* race s | NC_001329 |
| Glomerellales | *Verticillium dahlia* 76 | NC_008248 |
| Capnodiales | Mycosphaerella graminicola | NC_010222 |
| Pleosporales | *Phaeosphaeria nodorum* SN15 | NC_009746 |
| Basidiomycota | | |
| Microbotryales | *Microbotryum cf. violaceum* BFL-2013 | NC_020354 |
|  | *Microbotryum lychnidis-dioicae* strain MvSl135HT1 | NC_020353 |
| Agaricales | Moniliophthora perniciosa | NC_005927 |
|  | Moniliophthora roreri | NC_015400 |
| Cantharellales | Cantharellus cibarius | NC_020368 |
| Tilletiales | *Tilletia indica* F11 | NC_009880 |
|  | *Tilletia walker* TJ23 | NC_010651 |
| Polyporales | *Trametes cingulata* ATCC 26747 | NC_013933 |
| Ustilaginales | Ustilago maydis | NC_008368 |
| Basal lineages | | |
| Mucorales | *Rhizopus oryzae* DAOM 148428 | NC_006836 |
